# Lobulated esophageal schwannoma resected with concurrent approach from the thorax and cervix

**DOI:** 10.1186/s12957-018-1334-5

**Published:** 2018-02-13

**Authors:** Yoshinori Iwata, Chihiro Tanaka, Shuji Komori, Narutoshi Nagao, Masahiko Kawai, Kazuhiro Yoshida, Katsuyuki Kunieda

**Affiliations:** 1 Department of Surgery, Gifu Prefectural General Medical Center, 4-6-1 Noisshiki, Gifu, Japan; 2 Department of Surgical Oncology, Gifu University School of Medicine, 1-1 Yanagido, Gifu, Japan

**Keywords:** Esophageal schwannoma, Esophageal submucosal tumor, Mediastinal tumor, Positron emission tomography, Thoracotomy

## Abstract

**Background:**

Esophageal schwannomas are rare esophageal submucosal tumors. We herein report a case of a lobulated esophageal schwannoma resected with concurrent approach from the thorax and cervix.

**Case presentation:**

A 74-year-old woman visited our hospital with complaint of loss of consciousness, and a lobulated mediastinal tumor was discovered by chance in computed tomography. Upper gastrointestinal endoscopy showed a smooth elevated lesion at a position of 23–28 cm from the incisor teeth. A hypermetabolic appearance was noted on positron emission tomography. Based on these data, a gastrointestinal stromal tumor was suspected. The tumor was enucleated at the thoracic cavity while being pushed from the cervical incision. Pathological examination showed an esophageal schwannoma.

**Conclusions:**

We experienced a case of lobulated esophageal schwannoma with fluorodeoxyglucose accumulation. We resected the tumor with concurrent approach from the thorax and cervix.

## Background

Most esophageal tumors are cancer including squamous cell carcinoma and adenocarcinoma, and benign tumors are less than 1% of all esophageal tumors [1]. Leiomyoma is more than half of all benign esophageal tumors, and schwannoma is rare [1, 2]. The most common treatment for esophageal submucosal tumor is enucleation by surgery or endoscopy [3]. Recently, the cases of esophageal submucosal tumor resected by thoracoscopy have increased [4]. But the most important point in surgery is safety and curability, so we have to select which approach is better thoracoscopy surgery or thoracotomy by size of the tumor. We herein experienced a case of esophageal schwannoma resected with concurrent approach from the thorax and cervix.

## Case presentation

**Fig. 1:**
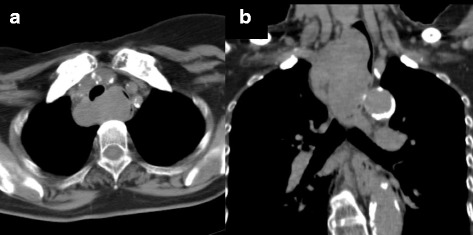
Computed tomography (CT). **a**, **b** A chest CT showed a lobulated mediastinum tumor extending to the cervix

**Fig. 2:**
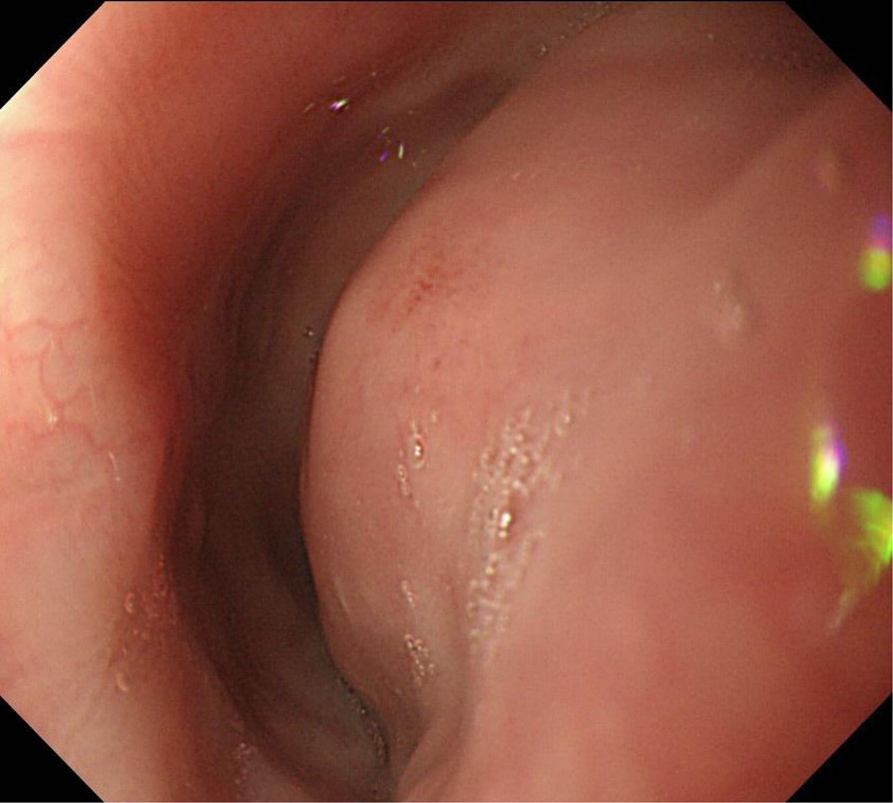
Upper gastrointestinal endoscopy. Upper gastrointestinal endoscopy showed a smooth elevated lesion

**Fig. 3:**
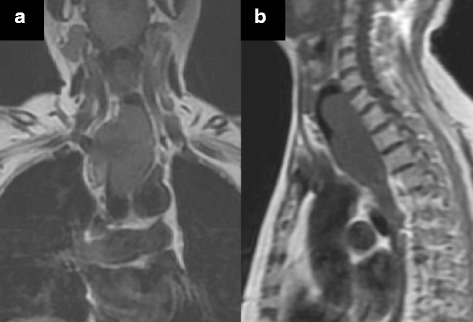
Magnetic resonance imaging. **a**, **b** MRI showed uniformity and clear boundary tumor

**Fig. 4:**
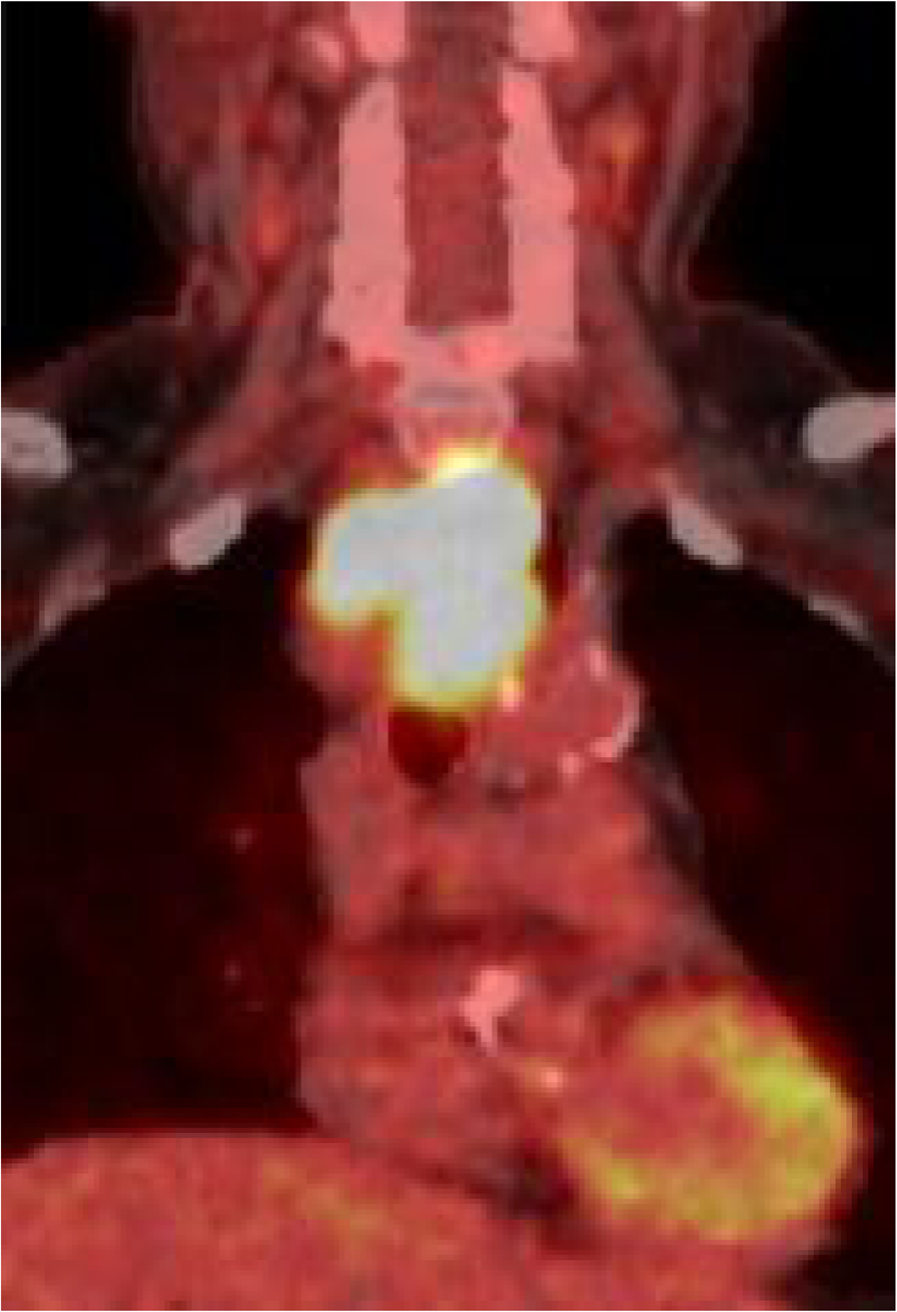
Positron emission tomography. Positron emission tomography showed hypermetabolic appearance

**Fig. 5:**
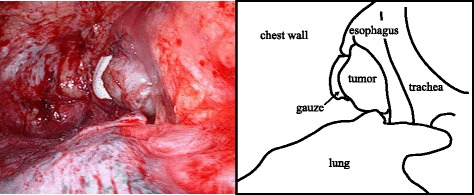
Intraoperative picture. The tumor was pushed by the gauze inserted from cervical incision

**Fig. 6:**
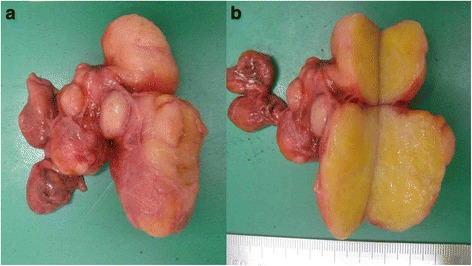
Surgical specimen. **a** The specimen showed a well-demarcated, elastic hard, and lobulated appearance and was measured 80 × 42 mm. **b** The cut surface was almost uniformly pale yellow

**Fig. 7:**
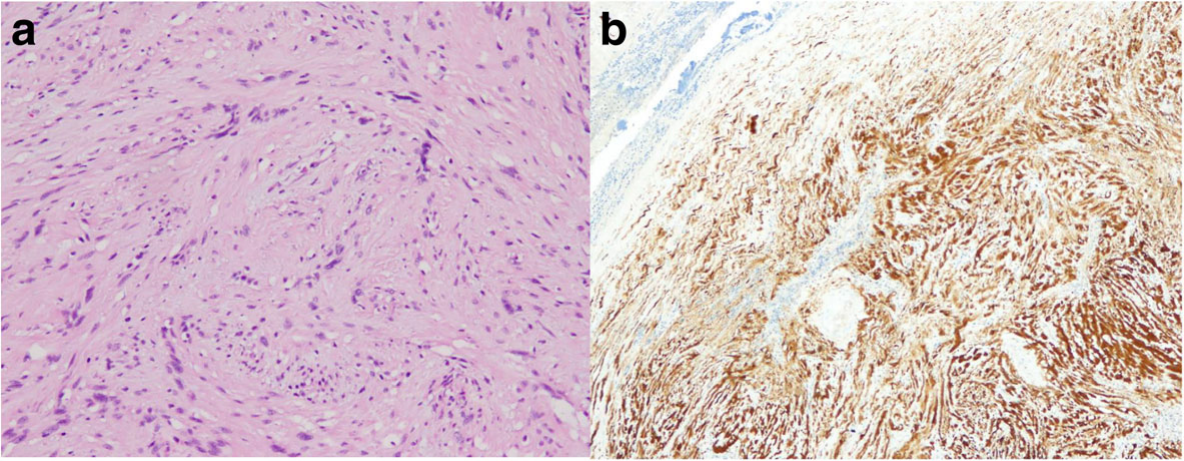
Histopathological examination. **a** Hematoxylin and eosin staining revealed spindle-shaped cells configuring plexiform proliferation. **b** Immunohistochemical examination revealed S-100 protein positivity

A 74-year-old woman visited our hospital with complaint of loss of consciousness, and a mediastinal tumor was discovered by chance in computed tomography (CT). A chest CT showed a lobulated tumor with a maximum diameter of 8 cm, which was located at upper mediastinum extending to cervix and was compressing the esophagus (Fig. 1a, b). Upper gastrointestinal endoscopy showed a smooth elevated lesion at a position of 23–28 cm from the incisor teeth (Fig. 2). Magnetic resonance imaging (MRI) showed uniformity and clear boundary tumor (Fig. 3a, b). A hypermetabolic appearance (maximum standardized uptake value, 15.0) was noted on positron emission tomography (PET) (Fig. 4). Based on these data, a gastrointestinal stromal tumor was suspected. The patient was placed in the left lateral position and underwent anterolateral thoracotomy via the sixth right intercostal space. At the same time, the skin incision was added in the right side of the cervix. The tumor was enucleated at the thoracic cavity while being pushed from the cervical incision (Fig. 5). The muscular layer of esophagus was repaired with sutures. The operation time was 245 min, and the blood loss was 551 g. The specimen showed a well-demarcated, elastic hard, and lobulated appearance and was measured 80 × 42 mm (Fig. 6a). The cut surface was almost uniformly pale yellow (Fig. 6b). Hematoxylin and eosin staining revealed spindle-shaped cells configuring plexiform proliferation (Fig. 7a). Immunohistochemical examination revealed S-100 protein positivity (Fig. 7b) and c-kit, α-smooth muscle actin, desmin, and CD34 negativity, establishing the diagnosis of esophageal schwannoma. The MIB-1 labeling index was < 10%. Her postoperative course was uneventful, and no recurrences have seen for 5 years after surgery.

## Discussion

Most esophageal tumors are cancer including squamous cell carcinoma and adenocarcinoma, and benign tumors are less than 1% of all esophageal tumors [1]. Leiomyoma is more than half of all benign esophageal tumors, and schwannoma is rare [1, 2]. Esophageal schwannoma more frequently develops in women than in men, and these tumors are often located in the upper and mid-esophagus in the mediastinum [5]. Some patients with esophageal schwannoma complain a variety of symptoms including dysphagia, dyspnea, chest pain, and coughing [6, 7]; others complain no symptom until the tumors grow larger.

Fluorodeoxyglucose (FDG)-PET as well as CT and MRI are reportedly useful for the confirmation of mediastinal tumors. FDG-PET is usually used to predict the malignancy potential of the tumor or to confirm the recurrence site of the cancer. Many cases of esophageal gastrointestinal stromal tumor with FDG accumulation are reported [8, 9]. In our case, we diagnosed esophageal gastrointestinal stromal tumor for the reason of esophageal submucosal tumor with FDG accumulation. Meanwhile, some esophageal schwannomas with FDG accumulation are reported [10–12]. Schwannomas originate from nerve cells that express glucose transporter type 3, and FDG uptake is considered to be increased for this reason [13]. Preoperative diagnosis of esophageal submucosal tumor is difficult with only imaging findings. On the other hand, EUS-FNA might be able to provide a diagnosis. In general, histological features of schwannoma include spindle-shaped tumor cells arranged in a palisading pattern or with loose cellularity in a reticular array. Immunohistochemical stainings are also useful as schwannoma shows S-100 protein positivity [14]. Although we thought we should undergo EUS-FNA because the huge mediastinal tumor with high FDG uptake had malignancy potential, the patient desired preserving esophagus regardless of the result of the EUS-FNA.

**Fig. 8:**
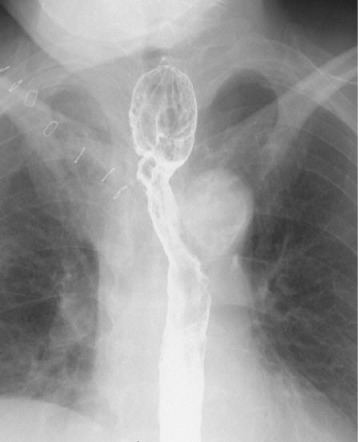
Upper gastrointestinal series. Upper gastrointestinal series showed no leakage, slight stenosis in the upper thoracic esophagus, and dilatation in cervical esophagus

Chemotherapy and radiation therapy are ineffective, and the most common treatment is enucleation by surgery or endoscopy for esophageal schwannoma [3]. In general, thoracoscopic resection is advantageous because it is less invasive, therefore shortening the hospital stay and reducing pain at the surgical wound site [15]. A retrospective study showed that minimally invasive resection of benign esophageal tumors is technically safe and associated with a shorter length of stay compared with open approaches [4]. Although no specific cutoff for size could be identified, most tumors greater than 7 cm were removed by thoracotomy [4]. In our case, we selected thoracotomy because of the size and shape of the tumor. In addition, as the tumor extended to the cervix, we thought it was difficult to resect the lobulated tumor only from the thorax. We try a concurrent approach from the thorax and cervix to secure the safety and curability. We supposed pushing the tumor from the cervix helped deciding the boundary of the tumor. Actually, the tumor was enucleated at the thoracic cavity while being pushed from the cervical incision with the patient placed in the left lateral position (Fig. 5). We needed an interrupting suture for the defect of muscular layer of esophagus and placed a chest drain near the suture. We removed the chest drain on postoperative day (POD) 6. We performed upper gastrointestinal series (Fig. 8) on POD 9 and found no leakage, slight stenosis in the upper thoracic esophagus, and dilatation in cervical esophagus. We started feeding on POD 10 and found no difficulty in swallowing. We thought endoscopy in the early postoperative period is not needed if the patient has no difficulty in swallowing. The patient was discharged without complications on POD 17. About the follow-up schedule for benign esophageal schwannoma, there has been no consensus yet. We have to follow the patient carefully because of the possibility of recurrence. We followed the patient with CT every 6 months and with endoscopy every year. No recurrences have seen for 5 years after surgery.

## Conclusions

We experienced a case of lobulated esophageal schwannoma with FDG accumulation. We resected the tumor with concurrent approach from the thorax and cervix.
